# Comparison of Vaccine Acceptance Between COVID-19 and Seasonal Influenza Among Women in China: A National Online Survey Based on Health Belief Model

**DOI:** 10.3389/fmed.2021.679520

**Published:** 2021-06-04

**Authors:** Liyuan Tao, Ruitong Wang, Jue Liu

**Affiliations:** ^1^Research Center of Clinical Epidemiology, Peking University Third Hospital, Beijing, China; ^2^Department of Epidemiology and Biostatistics, School of Public Health, Peking University, Beijing, China; ^3^National Health Commission Key Laboratory of Reproductive Health, Peking University Health Science Center, Beijing, China; ^4^Peking University Institute for Global Health and Development, Peking University, Beijing, China

**Keywords:** COVID-19, influenza, reproductive women, associated factors, vaccine acceptance

## Abstract

**Background:** Influenza could circulate in parallel with COVID-19. Studies focusing on the comparison of vaccine acceptance between COVID-19 and seasonal influenza are lacking. The aim of the study was to assess and compare vaccine acceptance of COVID-19 and influenza among reproductive women in China, in order to better understand and address factors associated with vaccine acceptance and to provide guidance for targeted measures to promote vaccination.

**Methods:** A national anonymous cross-sectional survey on COVID-19 and influenza vaccine acceptance among reproductive women aged 18–49 years in China was conducted online based on health belief model, a model widely used to evaluate health beliefs. Sociodemographic characteristics, health status, knowledge, attitude, and health beliefs related to COVID-19 and influenza infection and vaccination were retrieved. Pearson's χ^2^ test was used to compare the vaccine acceptance by the factors mentioned above. Multivariable logistic regression was used to assess the adjusted associations of factors related to vaccine acceptance. Paired *t*-test was used to compare scores of health beliefs between influenza and COVID-19 vaccinations.

**Results:** COVID-19 vaccine acceptance rate among reproductive women was 90.3% (95% CI 89.2–91.3%), which was significantly higher than influenza vaccine acceptance rate (85.5%, 95% CI: 84.2–86.7%). Influenza and COVID-19 vaccine acceptance both had the trends to decrease with age (all *p* < 0.05). Living in the western region, young age, a high level of knowledge scores on disease and vaccines, a high level of perceived susceptibility, a high level of cues to action, and a low level of perceived barriers were positively associated with both COVID-19 and influenza vaccine acceptance (all *p* < 0.05), while influenza vaccination history was additionally associated with influenza vaccine acceptance (*p* < 0.05).

**Conclusions:** Our findings suggest that tailored public health measures are needed to improve reproductive women's knowledge of COVID-19, influenza, and vaccines to alleviate women's vaccine hesitancy and expand vaccine uptake.

## Introduction

The coronavirus disease 2019 (COVID-19) is an acute respiratory infectious disease that has become a global pandemic, imposing tremendous burden on global health and severely disrupting the society and worldwide economics. The mortality and morbidity of COVID-19 have reached a record high of 110 million cases of infection and 2 million cases of death (1). The annual outbreak of seasonal influenza is also a major cause of worldwide mortality and morbidity, which remains a significant public health threat. The co-circulation of specific strains of influenza virus and inadequate influenza vaccination rates pose burden on healthcare systems (2).

Mass vaccination has long been regarded as the most effective approach to combat infectious diseases and curtail pandemic (3, 4). In the absence of effective therapy against COVID-19, safe and effective COVID-19 vaccines are imperatively called for halting the spread of coronavirus, recovering social economics from continuous disruption, and eventually establishing herd immunity. As both influenza and COVID-19 are caused by respiratory pathogens that share similar routes of transmission, along with the regular seasonality of influenza circulation (5, 6), specialists are concerned that influenza could circulate in parallel with COVID-19 in winter and spring. The co-infection of influenza and COVID-19 could emerge rapidly, bringing extra pressure to healthcare services and utilizing more medical resources during the strained period of public health emergencies. Thus, influenza and COVID-19 vaccinations are particularly essential for disease prevention, especially in the context of COVID-19 when extensive evidence suggests that influenza vaccination provides additional health and economic benefits to COVID-19 (7). Influenza vaccination reduces the risk of potential COVID-19 infection caused by hospital visits (8), thereby alleviating healthcare systems from the burden of treating both COVID-19 and influenza patients (6). Influenza vaccination also enhances the specificity of syndromic COVID-19 surveillance, as influenza and COVID-19 present similar symptoms (8). Hence, influenza and COVID-19 vaccinations should be a public health priority in the era of COVID-19.

As is shown in the draft landscape and tracker of COVID-19 vaccines provided by the World Health Organization (WHO), there were 70 vaccine candidates that have entered clinical development stage (9). Meanwhile, influenza vaccines have been recommended by public health authorities to vulnerable populations such as pregnant women and the elderly (10). Therefore, the broad acceptance of vaccination is crucial for relevant organizations to successfully pilot vaccination program and effectively curb the pandemic. However, extant literature has observed influenza and COVID-19 vaccine hesitancy among different populations (11). Vaccine hesitancy was defined as “delay in acceptance or refusal of vaccines despite availability of vaccine services” (12), which was listed by the WHO as one of the 10 threats to global health in 2019 (13). Vaccine hesitancy is complex and context specific, varying across time, places, and vaccines (14). It reflects people's vaccination intention and behavior, and it supports the design and evaluation of tailored interventions that aim to improve vaccination coverage (14). A growing literature has shown that the concerns regarding the safety of rapidly developed vaccines (11), mistrust in biomedical research (15), lack of relevant knowledge (16, 17), fear of adverse events (18), and lack of recommendations (19) contribute to COVID-19 and influenza vaccine hesitancy. During the COVID-19 pandemic, it is critical to initiate targeted measures to address vaccine hesitancy and improve vaccine acceptance, to promote vaccination on a large scale.

Despite the WHO's recommendations of influenza vaccination to populations of high priority, present research has found a suboptimal vaccine coverage. In a study conducted in Singapore, influenza vaccine coverage rate among pregnant women was merely 9.8% in 2017, while nearly half of the unvaccinated women stated they were unlikely to get vaccinated (19). In America, the rate was 61.2% during the 2019/2020 season (20), which was lower than the Healthy People 2020 target of 80% (21). In New Zealand, only 21.7% of pregnant women received influenza vaccines from 2013 to 2018 (22). The global influenza vaccination coverage warrants to be vigorously improved in the future, particularly during the COVID-19 pandemic period. Regarding the acceptance of COVID-19 vaccines among general population, the acceptance rate was 91.3% in China (23), 77.6% in France (24), and 68.5% in the United States (25). Though the acceptance of COVID-19 vaccines was relatively high, there was still a sizeable minority with vaccine hesitancy that await to be addressed. The vaccine acceptance should also be further monitored as vaccination behavior through tailored measures and follow-up.

The health belief model (HBM) is a model constructed based on the assumption that people are likely to take disease prevention behaviors and interventions (e.g., vaccination) if there is sufficient motivation (e.g., the perception that they are susceptible to the disease, the disease is severe, the behavior is beneficial, and barriers are minimal) and cues to action (e.g., recommendations from family members and healthcare workers) (26). It has been adopted as a conceptual framework that was widely used to evaluate the beliefs and attitudes toward vaccines (e.g., influenza vaccines, COVID-19 vaccines, human papillomavirus vaccines, and hepatitis B vaccines) and explain and predict vaccination behaviors (17, 27–29).

COVID-19 is an emerging global pandemic related to substantial morbidity and mortality, which has a higher case fertility rate and incidence rate than seasonal influenza. Additionally, the overall effectiveness and safety of the newly developed COVID-19 vaccines remain to be fully evaluated based on real-world evidence, while the well-established influenza vaccines are more widely delivered and are demonstrated to have a relatively high effectiveness and safety. Therefore, a comparison between influenza and COVID-19 vaccine acceptance is necessary in the dual epidemic of COVID-19 and influenza. Recent literature has found that men have higher propensity of vaccine acceptance than women (30, 31), but consensus has not been reached and is yet to be further discovered. Due to the universal recognition that both influenza and COVID-19 vaccinations should be promoted as a central public health measure in the context of COVID-19 pandemic, women's important role in families and the society, and the sparseness of research on influenza and COVID-19 vaccine acceptance among women at reproductive age, a nationwide anonymous cross-sectional survey on COVID-19 and influenza vaccine acceptance among reproductive women aged 18–49 years in China was conducted online based on HBM. The primary objective of this study was to assess and compare the level of vaccine acceptance relating to COVID-19 and seasonal influenza vaccines. The second objective was to identify factors that associate with COVID-19 and influenza vaccine acceptance. This study could provide guidance for vigorous educational campaigns, policy initiatives, and novel measures to eliminate conspiracy beliefs, establish vaccine confidence, and promote vaccination.

## Methods

### Study Design, Participants, and Sampling

This study was a nationwide anonymous cross-sectional survey using a stratified random sampling method via an online survey company: Wen Juan Xing (Changsha Ranxing Information Technology Co., Ltd., Hunan, China).

Wen Juan Xing (32) is a specialized data science company established in 2006. Its database covers factual and well-characterized personal information (e.g., sex, region, and age) of over 2.6 million Chinese respondents, which enables us to conduct stratified random sampling, recruit target participants, and distribute questionnaires. The data of the questionnaires are recorded in the database and can be used for further analysis. Thus, the platform allows us to carry out a representative sample and has been used to collect data in cross-sectional studies to investigate people's attitudes by many researchers (23, 33, 34). The target participants for this study were the reproductive women in China, and the inclusion criteria were as follows: (1) women aged 18–49 years; (2) women who attended the survey during December 14, 2020 to January 31, 2021; and (3) voluntary agreement to participate in the present study. On the basis of the sample size estimation, a sample size of at least 3,000 participants was anticipated to be reached.

The participants were stratified randomly selected via the online survey platform (Wen Juan Xing). The sampling of participants was divided into three stages. First, the target participants were divided into three tiers by region (the eastern, central, and western regions), and two provinces were randomly selected from each region. Second, the sample size for each province was allocated in proportion to the population of each province according to China Statistical Yearbook 2020, which contains relevant statistics (35). Third, target participants were randomly selected according to the sample size requirements in the Wen Juan Xing sample database and were recruited via Wen Juan Xing online platform.

The study was approved by the Ethical Committee of Peking University Third Hospital (IRB00006761-M2020528) and conducted according to the Declaration of Helsinki. Informed consent was obtained from all participants.

### Sample Size Estimation

The PASS software 14.0 (NCSS LLC, Kaysville, UT, USA) was used to calculate the sample size. According to the previous studies, the rates of COVID-19 vaccine acceptance and seasonal influenza vaccine acceptance were 85 and 75%, respectively (17, 36). Considering that the rate of COVID-19 vaccine acceptance was 85% (*p* = 0.85), with the alpha set as 0.05 and the confidence interval width as 0.1p (0.085), the sample size was 2,242 when using the exact (Clopper–Pearson) method for calculation. Likewise, the sample size was 1,191, considering the rate of influenza vaccine acceptance. Since there would be some unqualified questionnaires in the research process, the sample size of this study was planned to be 3,000 participants.

### Measures

A structured self-administered online questionnaire was designed based on the definition of the dimensions of HBM and questions of previous studies that used HBM to assess vaccine acceptance of other vaccine-preventable diseases. The questionnaire included the following parts: (1) sociodemographic characteristics, (2) health status, (3) knowledge on COVID-19 and seasonal influenza infection, (4) attitude toward COVID-19 and seasonal influenza vaccinations, and (5) health beliefs related with COVID-19 and seasonal influenza infections and vaccinations.

Sociodemographic characteristics included age group, region, education, occupation, and monthly household income per capita. Health status included gravidity, parity, history of chronic disease, and history of influenza vaccination. Knowledge on COVID-19 and influenza included six aspects: source of infection, route of transmission, susceptible population, common symptoms, high-risk population for severe illness and death, and individual preventive measures for infection. For each question, if the correct answer was chosen, the respondent received 1 score; otherwise, they would receive 0 score. The total knowledge score was divided into three groups (low, moderate, and high) by tertiles.

The primary outcome was the attitude toward COVID-19 and seasonal influenza vaccination. If participants answered “yes” in the question “If a vaccine becomes available for you, would you be willing to get the COVID-19/seasonal influenza vaccine?,” they were then classified into the acceptance group.

A total of 18 HBM questions were developed, with COVID-19 and influenza vaccination each having nine questions. There were two questions that evaluated perceived susceptibility to infection for themselves and their family members, one question on perceived severity of infection, three questions for perceived barriers (vaccines' safety, effectiveness, and the possibility of infection after vaccination), one question for perceived benefits of vaccination (protective effect), and two questions for cues to action (recommendations from physicians and family members). Each question was answered on a three-point Likert scale (“very concerned or agree,” “concerned or not sure,” and “not concerned or disagree') and were assigned as 3, 2, and 1 scores, respectively. The participants were divided into three groups according to the summed score for each HBM dimension by tertiles, with the top 33.3% of the participants assigned as the “high” group, bottom 33.3% assigned as the “low” group, and middle ones assigned as the “moderate” group. Cronbach's alpha index for different dimensions of the questionnaire ranged from 0.768 to 0.818 in seasonal influenza vaccination and ranged from 0.773 to 0.868 in COVID-19 vaccination, which both showed an adequate internal consistency reliability. The content of HBM questions is shown in Supplementary File 1.

### Statistical Analysis

Descriptive statistics were performed to describe the sociodemographic characteristics and the acceptance rates of the COVID-19 and influenza vaccines, and 95% CI was calculated. Pearson's χ^2^ test was used to compare the vaccine acceptance rates by sociodemographic characteristics, health status, levels of knowledge factors, and levels of health beliefs. Distributions of vaccine acceptance of COVID-19 and seasonal influenza by age groups were described in column chart. The Cochran–Armitage test for trend was used for examining the trend of proportion of the acceptance of COVID-19 and influenza vaccines by characteristics.

The multivariable logistic regression model was used to assess the adjusted associations of factors related to the acceptance of COVID-19 and influenza vaccines and was adjusted by region, age group, educational level, monthly household income per capita, gravidity, parity, chronic disease, history of influenza vaccination, score levels of knowledge on disease and vaccine, and score levels of five HBM dimensions. Adjusted odds ratios with 95% CIs for each variable were calculated. Paired *t*-test was used to compare the scores of HBM dimensions between COVID-19 vaccination and influenza vaccination. All the data analyses were conducted by SAS (version 9.4; SAS Institute, Cary, NC, USA), and two-sided *p* < 0.05 was considered statistically significant.

## Results

### Characteristics of the Study Population

In total, the response rate was 98.0%, with 3,150 participants out of 3,213 recruited participants completing the questionnaire. In the next stage, 139 participants were additionally excluded; and their data were cleaned up due to the extremely short time to fill in the questionnaire (<1 min). Ultimately, a total of 3,011 eligible women of reproductive age were included (Table 1). Among them, 1,804 (59.9%) participants acquired an education equivalent to or higher than bachelor's degree, 1,838 (61.0%) were 30 years old or younger, 1,607 (53.4%) participants had no prior experience of pregnancy, and 121 (4.0%) currently had chronic diseases including cardiovascular disease, cancer, diabetes, hypertension, and respiratory diseases. It is notable that only 833 (27.7%) of them had the history of influenza vaccination.

**Table 1:** Vaccine acceptance of COVID-19 and seasonal influenza among reproductive women aged 18–49 years in China by demographic characteristics.

	**Number of participants**	**Vaccine acceptance of COVID-19**			**Vaccine acceptance of seasonal influenza**		
		**Vaccine acceptance (%)**	**95% CI (%)**	***p*-value**	**Vaccine acceptance (%)**	**95% CI (%)**	***p*-value**
Region				<0.001			<0.001
Eastern	920	797 (86.6)	84.4–88.8		728 (79.1)	76.5–81.8	
Central	1,245	1,136 (91.2)	89.2–93.3		1,083 (87.0)	85.1–88.9	
Western	846	785 (92.8)	91.0–94.5		764 (90.3)	88.3–92.3	
Age group (years)				<0.001			<0.001
≤20	543	508 (93.6)	91.5–95.6		497 (91.5)	89.2–93.9	
21–25	712	666 (93.5)	91.7–95.3		643 (90.3)	88.1–92.5	
26–30	583	524 (89.9)	87.4–92.3		501 (85.9)	83.1–88.8	
31–35	469	404 (86.1)	83.0–89.3		390 (83.2)	79.8–86.5	
36–40	322	288 (89.4)	86.1–92.8		263 (81.7)	77.5–85.9	
41–45	207	180 (87.0)	82.4–91.5		155 (74.9)	69.0–80.8	
>45	175	148 (84.6)	79.2–89.9		126 (72.0)	65.3–78.7	
Education				<0.001			<0.001
Less than high school	321	272 (84.7)	80.8–88.7		262 (81.6)	77.4–85.9	
High school or some college	886	793 (89.5)	87.5–91.5		742 (83.7)	81.3–86.2	
Bachelor's degree	1,651	1,520 (92.1)	90.8–93.4		1,452 (87.9)	86.4–89.5	
Postgraduate degree	153	133 (86.9)	81.6–92.3		119 (77.8)	71.2–84.4	
Monthly household income per capita (RMB)				0.692			0.002
≤3,000	1,562	1,425 (91.2)	89.8–92.6		1,368 (87.6)	85.9–89.2	
3,001–5,000	693	622 (89.8)	87.5–92.0		587 (84.7)	82.0–87.4	
5,001–10,000	571	508 (89.0)	86.4–91.5		473 (82.8)	79.7–85.9	
>10,000	185	163 (88.1)	83.4–92.8		147 (79.5)	73.6–85.3	
Gravidity				0.002			<0.001
0	1,607	1,476 (91.8)	90.5–93.2		1,428 (88.9)	87.3–90.4	
1	624	561 (89.9)	87.5–92.3		514 (82.4)	79.4–85.4	
≥2	780	681 (87.3)	85.0–89.6		633 (81.2)	78.4–83.9	
Parity				0.002			<0.001
0	1,624	1,494 (92.0)	90.7–93.3		1,444 (88.9)	87.4–90.4	
1	825	730 (88.5)	86.3–90.7		669 (81.1)	78.4–83.8	
≥2	562	494 (87.9)	85.2–90.6		462 (82.2)	79.0–85.4	
Chronic disease				0.004			0.002
Yes	121	100 (82.6)	75.9–89.4		92 (76.0)	68.4–83.6	
No	2,890	2,618 (90.6)	89.5–91.7		2,483 (85.9)	84.6–87.2	
History of influenza vaccination	0.006			<0.001			
Yes	833	772 (92.7)	90.9–94.4		781 (93.8)	92.1–95.4	
No	2,178	1,946 (89.3)	88.1–90.6		1,794 (82.4)	80.8–84.0	
Total	3,011	2,718 (90.3)	89.2–91.3		2,575 (85.5)	84.3–86.8	

### Vaccine Acceptance by Demographic Characteristics

**Figure 1:**
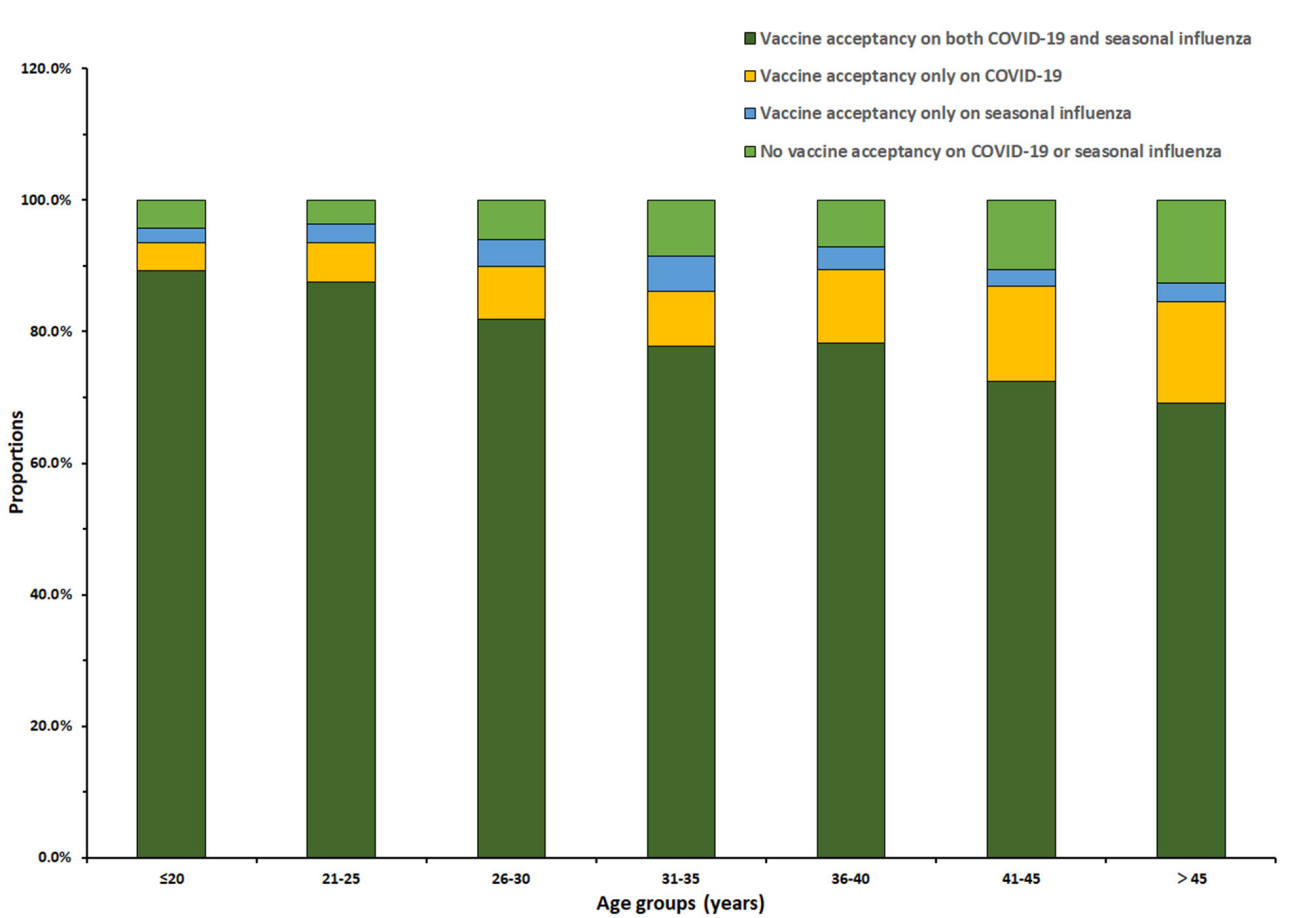
Distribution of seasonal influenza and COVID-19 vaccine acceptance by age groups.

**Figure 2:**
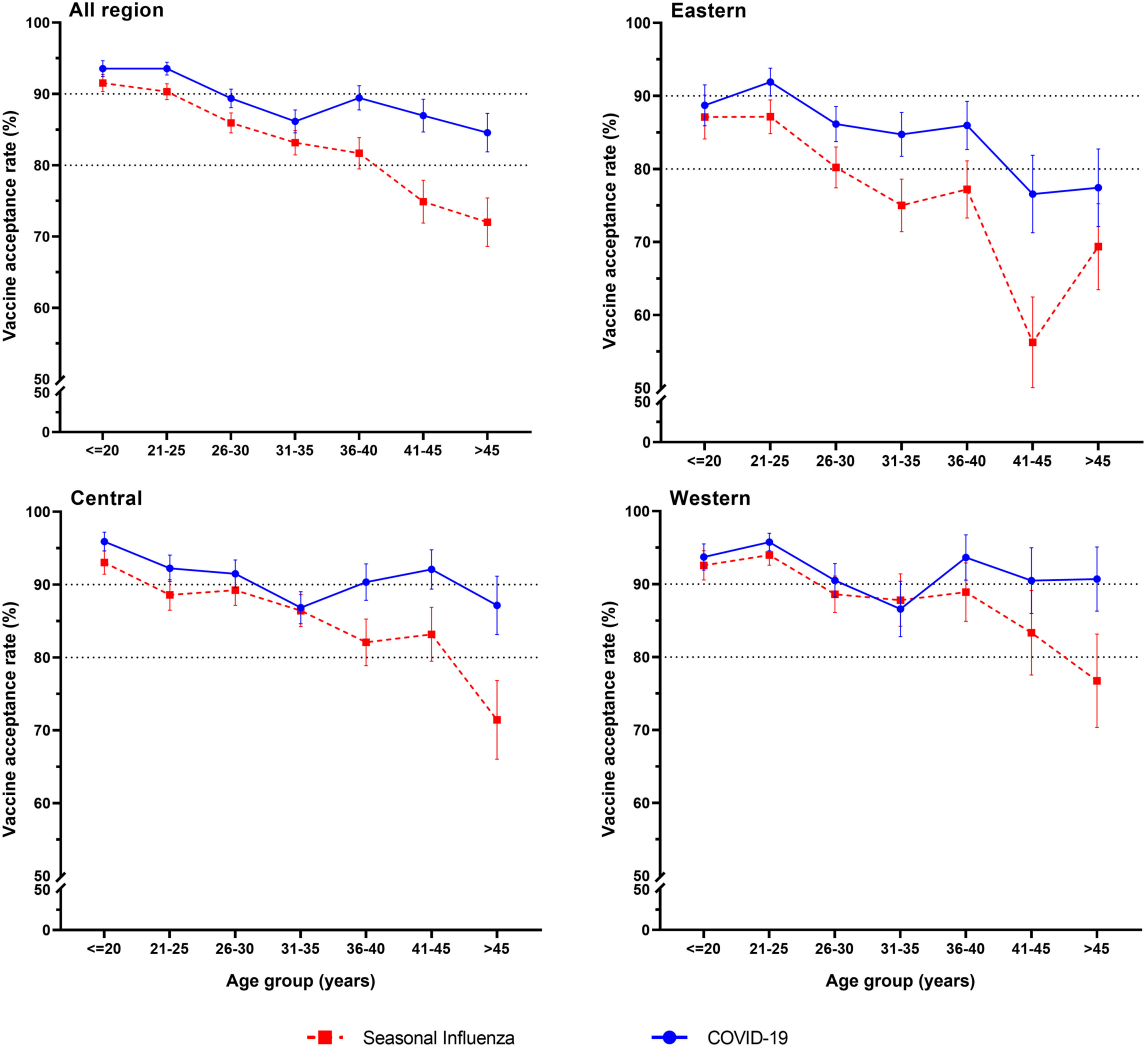
COVID-19 and seasonal influenza vaccine acceptance rates by age groups and regions.

Among the 3,011 reproductive women enrolled in our study, COVID-19 vaccine acceptance rate was 90.3 (95% CI: 89.2–91.3%), which was significantly higher than that of influenza vaccine (85.5%, 95% CI: 84.3–86.8%, p < 0.001). In total, the acceptance rate of only COVID-19 vaccines was 8.1% (95% CI: 7.2–9.1%), the acceptance rate of only influenza vaccine was 3.4% (95% CI: 2.7–4.0%), and the acceptance rate of both COVID-19 and influenza vaccines was 82.1% (95% CI: 80.8–83.5%) (Figure 1). Influenza vaccine and COVID-19 vaccine acceptance both had the trends to decrease with age (*p* < 0.001, Table 1), and the rate of COVID-19 vaccine acceptance was higher than that of the influenza vaccine for all age groups. For different regions, the eastern region had lower rates of vaccine acceptance, the acceptance rates of COVID-19 vaccine were <90%, and the acceptance rates of influenza vaccine were <80% for most age groups. The western region had the highest vaccine acceptance rates in all regions, with rates of more than 90% for the COVID-19 vaccine and more than 80% for influenza vaccine in most age groups (Figure 2).

Vaccine acceptance of COVID-19 and seasonal influenza among reproductive women in China by demographic characteristics is shown in Table 1. Regions, age groups, chronic disease, educational levels, gravity, and parity were associated with COVID-19 vaccine acceptance (*p* < 0.05, Table 1). In terms of influenza vaccine acceptance, monthly household income and history of influenza vaccination were additional associated factors (*p* < 0.05, Table 1). Those with history of influenza vaccination, with no child or prior experience of pregnancy, had a higher prevalence of influenza vaccine acceptance.

### Vaccine Acceptance by Knowledge and Health Belief Model Dimensions

Women with either influenza or COVID-19 vaccine acceptance presented higher levels of knowledge scores on disease and vaccines (all *p* < 0.001, Table 2). Regarding HBM dimensions, women with COVID-19 vaccine acceptance had significantly higher levels of perceived susceptibility, severity, benefit, and cues to action while having lower perceived barriers than those without COVID-19 vaccine acceptance (all *p* < 0.001). Women with influenza vaccine acceptance had significantly higher levels of perceived benefit, susceptibility, and cues to action while having lower perceived barriers than those without influenza vaccine acceptance (all *p* < 0.001).

**Table 2:** Vaccine acceptance of COVID-19 and seasonal influenza among reproductive women aged 18–49 years in China by knowledge and HBM dimensions.

	**Vaccine acceptance of COVID-19**			**Vaccine acceptance of seasonal influenza**		
	**Vaccine acceptance (%)**	**95% CI (%)**	***p*-value**	**Vaccine acceptance (%)**	**95% CI (%)**	***p*-value**
Score of knowledge^a^			<0.001			<0.001
Low	1,025 (87.2)	85.3–89.1		982 (83.6)	81.4–85.7	
Moderate	854 (91.7)	90.0–93.5		796 (85.5)	83.2–87.8	
High	836 (92.4)	90.6–94.1		797 (88.1)	86.0–90.2	
Perceived susceptibility			<0.001			0.001
Low	690 (86.5)	84.1–88.8		641 (80.3)	77.6–83.1	
Moderate	1,044 (89.6)	87.9–91.4		999 (85.8)	83.7–87.8	
High	981 (93.6)	92.1–95.1		935 (89.2)	87.3–91.1	
Perceived severity			0.001			0.097
Low	138 (84.1)	78.6–89.7		138 (84.1)	78.6–89.7	
Moderate	793 (88.2)	86.1–90.3		763 (84.9)	82.5–87.2	
High	1,784 (91.6)	90.3–92.8		1,674 (85.9)	84.4–87.5	
Perceived barriers			<0.001			<0.001
Low	980 (95.4)	94.1–96.7		927 (90.3)	88.4–92.1	
Moderate	750 (91.0)	89.1–93.0		696 (84.5)	82.0–86.9	
High	985 (84.9)	82.9–87.0		952 (82.1)	79.9–84.3	
Perceived benefit			<0.001			<0.001
Low	292 (83.7)	79.8–87.5		279 (79.9)	75.7–84.1	
Moderate	1,245 (87.9)	86.2–89.6		1,182 (83.4)	81.5–85.4	
High	1,178 (94.6)	93.4–95.9		1,114 (89.5)	87.8–91.2	
Cues to action			<0.001			<0.001
Low	655 (78.1)	75.3–80.9		642 (76.5)	73.7–79.4	
Moderate	809 (91.9)	90.1–93.7		770 (87.5)	85.3–89.7	
High	1,251 (96.8)	95.9–97.8		1,163 (90.0)	88.4–91.7	

*HBM, health belief model*.

a *The knowledge scores on the disease and the respective vaccines*.

### Comparison of Multivariate Factors Associated With Vaccine Acceptance on COVID-19 and Seasonal Influenza

The multivariate logistic regression analysis was adjusted by region, age group, educational level, monthly household income per capita, gravidity, parity, chronic disease, history of influenza vaccination, score levels of knowledge on disease and vaccine, and score levels of five HBM dimensions (perceived susceptibility, perceived severity, perceived barriers, perceived benefit, and perceived cues to action). The result has shown that the western region had higher vaccine acceptance than the eastern regions (Table 3), of which the acceptance of COVID-19 vaccine was more obvious than that of influenza vaccine (2.31 vs. 1.80 times). Older age (>25) had lower acceptance of both COVID-19 and influenza vaccines than younger age (≤20) (*p* < 0.05). The absence of influenza vaccination history decreased influenza vaccine acceptance (aOR = 0.34, 95% CI: 0.25–0.47) but was not associated with COVID-19 vaccine acceptance.

**Table 3:** Factors associated with vaccine acceptance on COVID-19 and seasonal influenza among reproductive women aged 18–49 years in China by multivariate logistic regression models.

**Factors**	**Vaccine acceptance of COVID-19**		**Vaccine acceptance of seasonal influenza**	
	**Adjusted odds ratio (95% CI)**	***p*-value**	**Adjusted odds ratio (95% CI)**	***p*-value**
Region		<0.001*		<0.001*
Eastern	1.00		1.00	
Central	1.75 (1.30, 2.35)	0.001	1.86 (1.45, 2.40)	<0.001*
Western	1.80 (1.27, 2.56)	<0.001*	2.31 (1.70, 3.13)	0.138
Age group (years)		<0.001*		<0.001*
≤20	1.00		1.00	
21–25	1.06 (0.66, 1.71)	0.854	0.81 (0.54, 1.23)	0.318
26–30	0.60 (0.38, 0.94)	0.027*	0.59 (0.39, 0.89)	0.026*
31–35	0.40 (0.26, 0.64)	<0.001*	0.42 (0.28, 0.64)	<0.001*
36–40	0.56 (0.33, 0.95)	0.040*	0.42 (0.27, 0.66)	<0.001*
41–45	0.54 (0.31, 0.95)	0.040*	0.30 (0.19, 0.49)	<0.001*
>45	0.39 (0.22, 0.70)	0.002*	0.27 (0.16, 0.44)	<0.001*
History of influenza vaccination			<0.001*	
Yes			1.00	
No			0.34 (0.25, 0.47)	<0.001*
Score of knowledge ^a^		0.041*		<0.001*
Low	1.00		1.00	
Moderate	1.44 (1.05, 1.96)	0.030*	1.61 (1.24, 2.11)	<0.001*
High	1.38 (1.01, 1.91)	0.050*	2.08 (1.56, 2.77)	<0.001*
Perceived susceptibility		<0.001*		<0.001*
Low	1.00		1.00	
Moderate	1.37 (1.01, 1.86)	0.041*	2.14 (1.65, 2.78)	<0.001*
High	2.21 (1.56, 3.12)	<0.001*	2.43 (1.81, 3.27)	<0.001*
Perceived barriers		<0.001*		<0.001*
Low	1.00		1.00	
Moderate	0.68 (0.45, 1.01)	0.059	0.70 (0.51, 0.97)	0.030*
High	0.36 (0.25, 0.52)	<0.001*	0.53 (0.39, 0.70)	<0.001*
Cues to action		<0.001*		<0.001*
Low	1.00		1.00	
Moderate	2.45 (1.80, 3.33)	<0.001*	2.46 (1.88, 3.23)	<0.001*
High	6.39 (4.41, 9.25)	<0.001*	4.19 (3.17, 5.54)	<0.001*

a *The knowledge scores on the disease and the respective vaccines. Factors that were not significantly associated with vaccine hesitancy are not shown in the table*.

High levels of knowledge scores on the disease and the respective vaccines, high levels of perceived susceptibility, and high levels of cues to actions were all positively associated with both the COVID-19 and influenza vaccine acceptance (all *p* < 0.05), while lower levels of perceived barriers were negatively associated with influenza and COVID-19 vaccine acceptance (*p* < 0.001).

### Comparison of Scores of Health Belief Model Dimensions

Regarding the comparison of scores of HBM dimensions (Table 4), women had higher scores of perceived susceptibility, lower perceived barriers, higher perceived severity, and higher cues to actions (all *p* < 0.05) of COVID-19 vaccination than influenza vaccination.

**Table 4:** Comparison of scores regarding the five dimensions of HBM between COVID-19 vaccination and seasonal influenza vaccination.

**HBM dimensions**	**Scores on COVID-19**	**Scores on seasonal influenza**	***p*-value**
Perceived susceptibility	4.14 ± 1.36	4.06 ± 1.23	<0.001
Perceived severity	2.59 ± 0.59	2.44 ± 0.66	<0.001
Perceived barriers	5.22 ± 1.46	5.30 ± 1.47	<0.001
Perceived benefit	2.30 ± 0.66	2.28 ± 0.68	0.093
Cues to action	5.02 ± 1.09	4.97 ± 1.11	0.003

*The paired t-test was used for the comparison between groups*.

*HBM, health belief model*.

## Discussion

To our best knowledge, this is the first study to assess and compare factors associated with COVID-19 and influenza vaccine acceptance among women of reproductive age. A nationwide anonymous cross-sectional survey of vaccine acceptance on COVID-19 and seasonal influenza among reproductive women aged 18–49 years in China based on HBM was conducted online via Wen Juan Xing platform to assess and compare vaccine acceptance between COVID-19 and seasonal influenza vaccination, and to understand factors associated with vaccine acceptance. This study aims to provide evidence for policy initiatives, educational campaigns, and novel approaches to reduce vaccine hesitancy and raise vaccination acceptance.

Our findings revealed several factors that had a significant impact on reproductive women's COVID-19 vaccine acceptance, including regions and educational level. History of influenza vaccination was additionally associated with influenza vaccine acceptance, which was generally consistent with the results of previous studies (37–39). A national survey carried out among women in France found that influenza vaccine uptake was associated with high educational level (37). In Scotland, Williams and his colleagues discovered that people with higher educational levels have higher intentions of accepting COVID-19 vaccine (38). High education level may lead to a better knowledge of diseases and vaccine and may relate to a lower probability to believe in conspiracies and fake news, thus raising women's awareness of the necessity of receiving vaccination and resulting in higher vaccination acceptance. During COVID-19 pandemic, the severity of the pandemic and influenza varied between regions. Therefore, women from different regions may have different recognition and attitudes toward the diseases and vaccines, accounting for the distant degrees of vaccine acceptance between regions. It is noteworthy that our finding suggests women from the eastern region had a lower influenza and COVID-19 vaccine acceptance than women from the western region. This is a worrying finding, for the eastern region had a far higher population density than the western region, which leads to a higher risk of virus transmission within the eastern region. Given the above conditions, low vaccine acceptance among might lead to extra heavy burden to the eastern region, which calls for timely publicity and an enhancement of vaccination mobilization in the eastern region. Prior influenza vaccination experience could reduce women's concerns about the safety and effectiveness of vaccine, thus contributing to their willingness of vaccine acceptance.

Many studies found that younger individuals had lower vaccine acceptance, which was the opposite of our finding (30, 31, 39). As aging is a predominant risk factor for severe disease and death from COVID-19 and influenza (40, 41), high-risk old populations should be targeted as the priority group to receive vaccination. However, influenza and COVID-19 vaccine acceptance were found to have the trends to decrease with age, which is a worrying finding. One speculation was that reproductive women with younger ages attained a larger amount of information from social media, schools, or workplace. Consequently, younger women tend to have more access to information and have a better knowledge of the severity of the diseases and the safety and effectiveness of vaccines, which contributed to their vaccine acceptance. Given that older people tend to use social media less often to access information than the younger, more offline targeted campaigns about vaccination are urgently needed in community and hospitals to inform older populations of their vulnerability and susceptibility to influenza and COVID-19, the severity of the diseases, and the benefits of vaccines.

COVID-19 vaccine acceptance rate was found to be significantly higher than influenza vaccine acceptance rate. Meanwhile, women in our study were found to have higher perceived severity regarding COVID-19 than influenza, which might lead to reproductive women's higher attention to COVID-19 prevention. However, influenza immunization is of critical importance and should be considered as a public health priority in the context of COVID-19, for it reduces the risk of co-infection with COVID-19 and increases the precision of COVID-19 diagnosis and management in terms of antiviral therapy and infection control, thereby alleviating the burden of healthcare systems. Therefore, relevant public health measures should also target influenza vaccination instead of focusing on COVID-19 vaccination alone.

Though influenza vaccine acceptance rate in our survey was relatively high, the uptake rate of influenza vaccine was only 27.7%. This may be due to the speculation that in the context of COVID-19, people tend to be more aware of the effectiveness of vaccines in the prevention of infectious diseases and attach great importance to vaccination. Thus, the level of influenza vaccine acceptance may increase due to the impact of COVID-19 compared with that in the past seasons. A study that evaluated the impact of COVID-19 pandemic on the uptake of influenza vaccines in the United Kingdom found that COVID-19 increased the influenza vaccination acceptance in previously eligible but unvaccinated people and motivated substantial uptake in newly eligible people (42), which indicated the impact of COVID-19 pandemic on promoting influenza vaccination. Another possible reason is that people's intention of influenza vaccination does not necessarily turn into vaccination behavior in the end; it may be influenced by other factors including the convenience and cost of vaccination. More in-depth researches are needed to further investigate the reasons for the inconsistency of vaccination intention and behavior, which is essential for public health workers and policymakers to take tailored measures to deal with the barriers and largely increase the rate of influenza vaccination. The uptake rate of influenza vaccines in the 2020–2021 season also awaits to be investigated to assess the impact of COVID-19 and the effect of relevant vaccination publicity.

As shown in the multivariate logistic regression analysis, both COVID-19 and influenza vaccine acceptance were associated with higher knowledge scores, higher perceived susceptibility and cues to action, and lower perceived barriers. These dimensions represent significant targets for future interventions in vaccine campaign. Public health professionals and relevant organizations should take full advantage of social media to disseminate relevant health information about influenza, COVID-19, and the vaccines, including people's susceptibility to the diseases, potential complications, and adverse effects caused by diseases. Relevant offline campaigns should also be put in place to inform reproductive women of the benefits, safety, and effectiveness of vaccines. As physicians and family members play an important role in raising women's vaccination acceptance, they should also be targeted and mobilized to encourage vaccine uptake in reproductive women.

Reproductive women constitute a large portion of COVID-19 high-risk populations such as healthcare personnel (43), which meet the population prioritized for vaccination. Furthermore, safe and effective COVID-19 vaccination for pregnant women is imperative to protect mothers, fetuses, and the newborns from serious illness. However, a vast majority of COVID-19 vaccine clinical trials specifically exclude pregnant women due to potential risks of vaccination in pregnant women and adverse effect of medical exposure among fetus, which results in limited data of the effectiveness and safety of vaccines. Currently, most organizations recommended that pregnant or lactating women may be vaccinated when the benefit is deemed to outweigh potential risk. A consultation with healthcare professionals is recommended but not required. Healthcare professionals should inform pregnant or lactating women of the absence of data on efficacy and safety specific to the vaccine use in pregnant women. More data and post marketing research of COVID-19 vaccines on risk–benefit assessment among pregnant or lactating women are needed.

The study has several limitations. First, this is a cross-sectional study that investigated women's vaccine acceptance and its associated factors, so the effect of tailored public health measures on vaccine acceptance could not be evaluated. Second, vaccine acceptance of pregnant women among the reproductive women recruited in our study was not specifically evaluated. Third, the questionnaire was released and completed when COVID-19 vaccines were not available, which might have a potential impact on reproductive women's vaccine acceptance. As more details regarding COVID-19 vaccine are known to the public, alternations may occur in people's attitudes toward vaccination. Last, since the questionnaire was distributed to the participants via an online platform, only the women who had access to the online platform were recruited to complete the questionnaire. Thus, the results may not be generalizable to all women in China.

## Conclusion

COVID-19 vaccine acceptance rate among reproductive women was 90.3%, which was significantly higher than influenza vaccine acceptance rate (85.5%). Influenza and COVID-19 vaccine acceptance both had the trends to decrease with age. Living in the western region, young age, a high level of knowledge scores, a high level of perceived susceptibility, a high level of cues to action, and a low level of perceived barriers were associated with both COVID-19 and influenza vaccine acceptance, while history of influenza vaccination was additionally associated with influenza vaccine acceptance. HBM is widely used to evaluate people's attitudes toward vaccines and predict vaccination behavior, which could be a framework for vaccine promotion and management. Our findings suggest that policymakers, professionals, and other researchers should take tailored public health measures to improve reproductive women's knowledge on diseases and vaccines, inform them of the severity and their susceptibility of COVID-19 and influenza, and recommend vaccination to them, to alleviate reproductive women's vaccine hesitancy, improve vaccine acceptance, and expand vaccine uptake.

## Data Availability Statement

The raw data supporting the conclusions of this article will be made available by the authors, without undue reservation.

## Ethics Statement

The study was approved by the Ethical Committee of Peking University Third Hospital and conducted according to the Helsinki Declaration (IRB00006761-M2020528). The patients/participants provided their written informed consent to participate in this study.

## Author Contributions

JL and LT contributed to conception and design of the study. LT and RW organized the database and wrote the first draft of the manuscript. LT, JL, and RW performed the statistical analysis and wrote sections of the manuscript. All authors contributed to manuscript revision and read and approved the submitted version.

## Conflict of Interest

The authors declare that the research was conducted in the absence of any commercial or financial relationships that could be construed as a potential conflict of interest.
