# Supplementary material for: Comparison of Vaccine Acceptance Between COVID-19 and Seasonal Influenza Among Women in China: A National Online Survey Based on Health Belief Model
Source: Front Med (Lausanne). 2021 Jun 4;8:679520. doi: 10.3389/fmed.2021.679520 (PMC8211886; doi:10.3389/fmed.2021.679520)
Supplement: Supplementary file 1 [file Table_1.docx]

**Supplemental file 1. Questions related to the Health Belief Model dimensions in the questionnaire**

**Perceived susceptibility:**

1. To which extent are you concerned about the following statements?

|  | Very concerned | concerned | Not concerned |
| --- | --- | --- | --- |
| I’m concerned about myself getting influenza. | □ | □ | □ |
| I’m concerned about my family members getting influenza. | □ | □ | □ |
| I’m concerned about myself getting COVID-19. | □ | □ | □ |
| I’m concerned about my family members getting COVID-19. | □ | □ | □ |

**Perceived severity**

|  | Agree | Not sure | Disagree |
| --- | --- | --- | --- |
| If a woman gets seasonal influenza, she is more likely to have severe illness. | ○ | ○ | ○ |
| If a woman gets COVID-19, she is more likely to have severe illness. | ○ | ○ | ○ |

**Perceived barriers**

| Seasonal influenza vaccination can cause a person to get sick with seasonal influenza. | ○ | ○ | ○ |
| --- | --- | --- | --- |
| Seasonal COVID-19 vaccination can cause a person to get sick with COVID-19. | ○ | ○ | ○ |
| Seasonal influenza vaccination is not safe. | ○ | ○ | ○ |
| COVID-19 vaccination is not safe. | ○ | ○ | ○ |
| Vaccine is not an effective way to prevent a woman from getting seasonal influenza. | ○ | ○ | ○ |
| Vaccine is not an effective way to prevent a woman from getting COVID-19. | ○ | ○ | ○ |

**Perceived benefits**

| Getting influenza vaccine is benefit for women. | ○ | ○ | ○ |
| --- | --- | --- | --- |
| Getting COVID-19 vaccine is benefit for women. | ○ | ○ | ○ |

**Cues to action**

| If a physician recommends influenza vaccination, I would get vaccinated. | ○ | ○ | ○ |
| --- | --- | --- | --- |
| If a physician recommends COVID-19 vaccination, I would get vaccinated. | ○ | ○ | ○ |
| If family members recommend influenza vaccination, I would get vaccinated. | ○ | ○ | ○ |
| If family members recommend COVID-19 vaccination, I would get vaccinated. | ○ | ○ | ○ |
